# The Organoid Platform: Promises and Challenges as Tools in the Fight against COVID-19

**DOI:** 10.1016/j.stemcr.2020.11.009

**Published:** 2021-03-09

**Authors:** Maarten H. Geurts, Jelte van der Vaart, Joep Beumer, Hans Clevers

**Affiliations:** 1 Hubrecht Institute, Royal Netherlands Academy of Arts and Sciences (KNAW) and University Medical Center Utrecht, 3584 CT Utrecht, the Netherlands; 2 Oncode Institute, Hubrecht Institute, 3584 CT Utrecht, the Netherlands

**Keywords:** organoid, SARS-CoV-2, COVID-19, virus

## Abstract

Many pathogenic viruses that affect man display species specificity, limiting the use of animal models. Studying viral biology and identifying potential treatments therefore benefits from the development of *in vitro* cell systems that closely mimic human physiology. In the current COVID-19 pandemic, rapid scientific insights are of the utmost importance to limit its impact on public health and society. Organoids are emerging as versatile tools to progress the understanding of SARS-CoV-2 biology and to aid in the quest for novel treatments.

## Teaser

In this work Beumer, Clevers, and colleagues review the current state of affairs of ASC- and iPSC-derived organoids in fighting the current SARS-CoV-2 pandemic. To limit the impact of this pandemic, rapid scientific insights are crucial. Organoids closely resemble human physiology, and are emerging for studying viral biology and to identify potential treatments for current and future pandemics.

## Main Text

The global outbreak of the novel coronavirus SARS-CoV-2 strains healthcare systems around the world and has already caused over a million deaths. COVID-19 is the third appearance of a lethal coronavirus, after the emergence of severe acute respiratory syndrome coronavirus (SARS-CoV) in 2002 and Middle East respiratory syndrome virus (MERS-CoV) in 2012. It appears likely that more novel viruses will emerge to infect humans on a global scale. To develop effective treatments for current and future infectious diseases, it is key to establish *in vitro* models that closely resemble the physiology of the viral host and that are likely to allow—and model—infection by any type of virus. Much of the insight on coronavirus biology has been gathered using “classical” 2D cell lines, such as the Vero E6 cell line, derived from the kidney of the African green monkey (Matsuyama et al., 2020). These cell lines are often easily infectable by viruses and—if so—can be used to propagate the virus for downstream experiments. However, cell lines are typically malignantly transformed and consist of a homogeneous population of poorly differentiated cells, which hampers culturing of “picky” viruses. As a case in point, Norovirus has remained unculturable on such cell lines as it requires a differentiated intestinal cell type as its target (Alvarado et al., 2018; Ettayebi et al., 2016). Also, the biology of infection and specific pathological effects seen in a patient's tissue may not be reproduced on these transformed cell lines. As an example, the pathogenic effects of Zika virus on the fetal human brain cannot be modeled using cell lines (Qian et al., 2017; Watanabe et al., 2017). Moreover, treatment efficacy may not be directly extrapolated to the clinic, as illustrated by the recent controversy around the use of the anti-malaria drug hydroxychloroquine. This drug was quickly adapted worldwide as a promising treatment against SARS-CoV-2 as it was shown to be effective in Vero cells (Wang et al., 2020a). However, besides countless side effects, hydroxychloroquine has now been proven to be ineffective in humans (Hernandez et al., 2020). In the search for the most optimal *in vitro* model, organoids are now emerging.

## Basics of Organoid Technology: The Adult and iPSC Flavors

Organoids are 3D structures grown from stem cells and consist of organ-specific cell types that self-organize through cell sorting and spatially restricted lineage commitment (Clevers, 2016; Lancaster and Knoblich, 2014). They can be established from either induced pluripotent stem cells (iPSCs) or multipotent adult tissue stem cells (ASCs). Under optimal culture conditions, stem cell differentiation generates a complete arsenal of cell types as present in the tissue of interest. Organoid models are genetically stable and can be expanded over long periods of time. Organoid derivation from iPSCs or ASCs differs greatly. iPSC-derived organoids are formed by first creating a 3D aggregate of iPSCs, a so-called embryoid body, after which organ and cell-type specialization is initiated by closely mimicking the developmental signals. This process can take multiple weeks, a time during which gastrulation and organogenesis are mimicked in a dish. Apart from the epithelial cell types produced, suboptimal specification of iPSCs can also lead to the production of non-epithelial lineages, such as fibroblasts and muscle. ASC-derived organoids are established by directly dissociating the tissue of interest, containing the intrinsic ASCs and propagating these cells over extended periods of time under tissue-specific growth factor conditions (van der Vaart and Clevers, 2020). ASC-derived organoids are now readily established from most murine and human epithelial tissues (Driehuis et al., 2020). Tissue-specific cell types can subsequently be enriched by modulating individual signaling pathways, such as Notch or BMP (Beumer and Clevers, 2020). The ability to generate a variety of human tissues in the form of organoids has become important in the study of COVID-19 when it was realized that patients present with systemic symptoms beyond the respiratory infection.

## Organoids Go Viral to Study Infection Diseases

The vast majority of airborne viruses, including respiratory and enteroviruses, enter the human body through infection of epithelial cells. Multiple studies have used ASC-derived organoids from the intestinal lining, oral mucosa, or airway as infection models for their respective viruses. Human oral mucosal organoids recapitulate the stratified architecture of the oral mucosa and have been shown to be receptive to infection by herpes simplex virus type 1 and human papilloma virus (Driehuis et al., 2019). Human intestinal organoids have become the first *in vitro* model system to study viral entry and replication of noroviruses (Ettayebi et al., 2016; Haga et al., 2020). Norovirus infectivity and replication efficiency differ greatly between individuals. Intestinal organoids from individuals indeed recapitulated the diverging norovirus replication efficiency *in vitro* (Estes et al., 2019). The pulmonary organoid epithelium is susceptible to a wide range of viruses. Sachs et al. (2019) used the 3D structure of organoids to identify increased cellular motility after infection with respiratory syncytial virus (RSV). This phenomenon was identified *in vivo* but could not be recapitulated and studied in traditional 2D cell lines or primary cells in air-liquid interface (ALI) cultures. Subsequent analysis of this increased cellular motility showed that overexpression of the RSV non-structural protein NS2 led to increased motility and fusion with uninfected organoids. This study thereby suggested an unappreciated role of cellular motility for viral propagation in the airway epithelium (Sachs et al., 2019). Airway organoids have also emerged as tools for influenza viruses that display species-restricted tropism. Zhou et al. (2018) showed that human airway organoids are readily infected with human infectious influenza virus H7N9 while avian H7N2 showed significantly lower replication levels. H1N1 caused a pandemic in 2009. H1N1 isolated from humans readily replicated in airway organoids while the same serotype virus isolated from swine showed much lower replication rates. Organoids could thus be used to study potential zoonotic events (Zhou et al., 2018). During the 2015 Zika virus epidemic, cerebral organoids derived from iPSCs were used to gain evidence that this virus selectively replicates in dividing neural progenitors (Qian et al., 2017; Watanabe et al., 2017). This work provided the explanation why the fetal human brain was severely affected, while the virus was less destructive to the postnatal brain. Many human viruses originate from animals and have undergone small adaptations before transmission to man. For example, multiple bat species are notorious carriers of zoonotic viruses that occasionally jump to humans, including the filoviruses Marburg and Ebola, and coronaviruses. ASC-derived intestinal organoids have been derived from non-human species, including bat and feline. This has allowed the propagation of strains of coronaviruses directly from bats (Tekes et al., 2020; Zhou et al., 2020). These efforts highlight how organoids can be exploited to study (species-specific) viruses with zoonotic potential. The ASC-derived organoid system is also translatable to non-mammalian species, such as reptilians, and could thus facilitate research into viruses affecting other parts of the animal kingdom (Post et al., 2020). In some areas this may be urgent: Python nidoviruses, for example, have become the primary cause of fatalities among pythons in captivity (Blahak et al., 2020). Moreover, organoids could also minimize the impact of studying multiples of the affected and endangered animals, such as pangolins and civets.

## Fighting the SARS-CoV-2 Pandemic with Organoids

**Figure 1:**
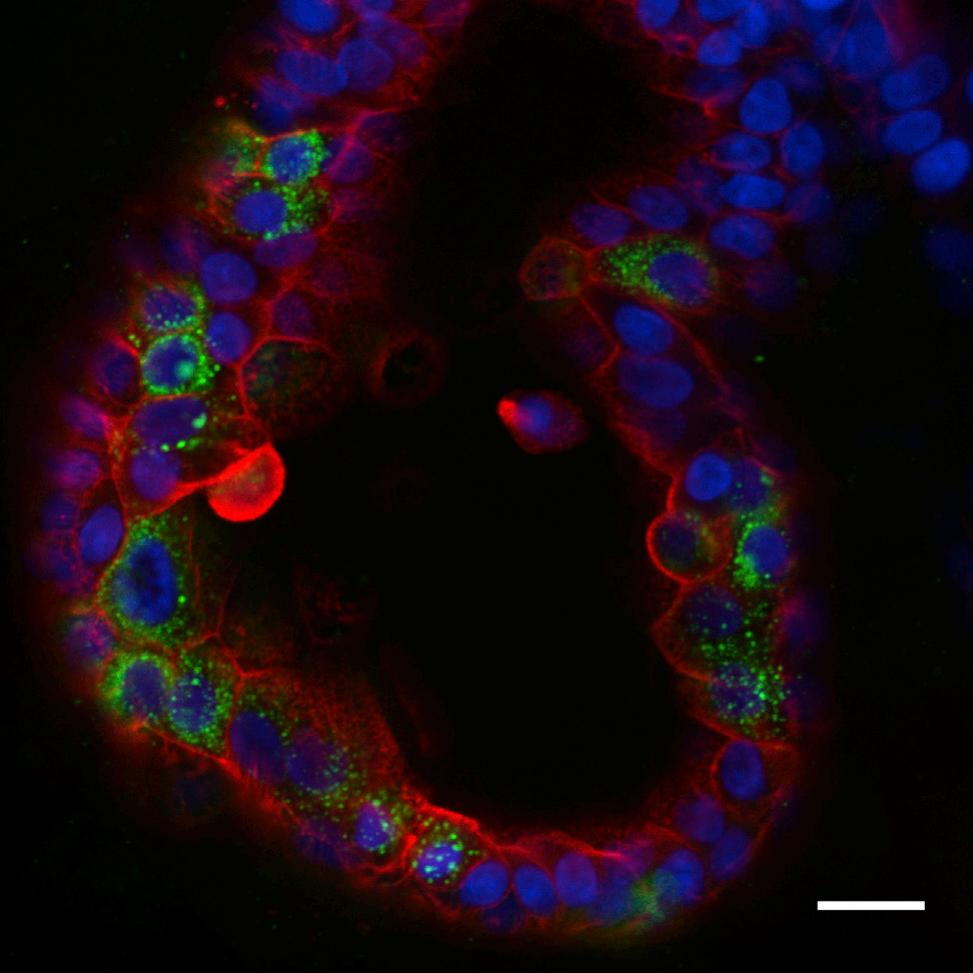
Confocal Image of a SARS-CoV-2 Infected Small Intestinal Organoid The organoid was stained for virus (anti-dsRNA, green), actin (phalloidin, red), and nuclei (DAPI, blue). Scale bar, 20 μm.

SARS-CoV-2 first emerged in December 2019 in the city of Wuhan in China and has led to more than 1 million deaths to date (WHO website). Like SARS, this new family member of the coronaviridae is mainly known for causing symptoms in the airways. Therefore, the virus has been studied using airway-derived culturing platforms: upper airway organoid-derived ALI cultures, as well as distal airway ASC organoids (Figure 1) (Lamers et al., 2020; Salahudeen et al., 2020). ALI cultures of adult upper airway-derived organoids were efficiently infected by addition of the virus to the apical side. SARS-CoV-2 infection was observed in ciliated cells, which further supported the claim that these cells represent the primary target of the virus. While these ALI cultures could be accessed from the apical side, the distal lung organoids reported by Salahudeen et al. (2020) needed to be “flipped” to generate an apical-out morphology before they could be infected by SARS-CoV-2. Within these cultures, contrary to previous studies, SARS-CoV-2 infected only goblet cells but not ciliated cells. The development of a feeder-free culture system for alveolar-like cells allowed the generation of an alveolar model for SARS-CoV-2 replication (Salahudeen et al., 2020). Apart from the dominating respiratory symptoms, gastrointestinal symptoms have been identified in a subset of patients (Gupta et al., 2020). To potentially elucidate another mode of transmission, human small intestinal organoids were infected by SARS-CoV-2 in three simultaneous studies. While one study used 2D monolayers generated from organoids to access the apical side, the two others made use of the 3D architecture and inoculated the organoids in suspension after their mechanical disruption. All three studies concluded that enterocytes of the intestinal lining were successfully infected by SARS-CoV-2 (Lamers et al., 2020; Zang et al., 2020; Zhou et al., 2020). These studies imply that the intestine is a potential site of SARS-CoV-2 replication, explaining gastrointestinal symptoms observed in patients. Due to the cellular complexity of these small intestinal organoids, insights in host tropism could be readily identified. Bulk mRNA sequencing of infected organoids reveal the response of the epithelium over time, with multiple cytokines and interferon-stimulated genes (ISGs) displaying different kinetics (Lamers et al., 2020). Furthermore, multiple iPSC-derived organoids have been employed to showcase SARS-CoV-2 replication and study its associated pathologies in tissues where ASC organoids are less developed. Ramani et al. (2020) showed that SARS-CoV-2 enters iPSC-derived cerebral organoids within 2 days, although no replication could be observed in neurons. Extensive cell death was seen in neurons, associated with altered distribution of the structural protein Tau. Monteil et al. (2020) used vascular and kidney organoids derived from iPSCs and found that these cells facilitated SARS-CoV-2 replication. They used these cultures to verify human recombinant soluble ACE2 as an inhibitor of infection of SARS-CoV-2. Together, the repertoire of ASC- and iPSC-derived organoids provides a valuable platform to study tissue-specific dynamics upon SARS-CoV-2 infection in relevant and physiologic models.

## The CRISPR Search for Host Factors of SARS-CoV-2 Entry

Much has been elucidated about the way SARS-CoV-2 infects host cells using converging efforts ranging from *in vitro* cell culture systems to crystallography. Currently it is thought that, as the virus is in the proximity of the cell, the protease TMPRSS2 cleaves the spike protein of SARS-CoV-2, upon which the virus enters the cells in an ACE2-dependent manner (Hoffmann et al., 2020). However, besides these two proteins many other host factors, such as TMPRSS4, DPP4, and ANPEP (the latter two are well known from other coronaviruses) have been implicated to play a role in the infection process. Therefore, to date the exact cell entry pathway of SARS-CoV-2 remains a crucial field of research (LoPresti et al., 2020). One way of deciphering this conundrum could be by performing loss-of-function screens. A loss-of-function screen is efficiently performed by generating knockout cell lines with the use of CRISPR-Cas9. Upon target recognition by a short guide RNA, the endonuclease Cas9 creates a double-strand break resulting in error-prone DNA repair. This results in formation of small insertions and deletions (indels). The consequent frameshifts lead to loss of function of the protein of interest (Mali et al., 2013). CRISPR was effectively utilized in organoids by Zang et al. (2020) to create population-wide knockouts of TMPRSS2 and TMPRSS4 in duodenum enteroids using a lentiviral-based system. Abrogation of TMPRSS4 led to a 4-fold reduction of SARS-CoV-2 replication, whereas TMPRSS2 knockout did not result in a significant reduction. CRISPR loss-of-function screens can furthermore be performed in a genome-wide manner to identify unknown host factors in an unbiased manner. By cloning a library of guide RNAs in a lentiviral vector, a cell library containing individual knockouts of all genes in the genome can be created. Genome-wide CRISPR screens have become an emerging technique for identifying genes involved in a plethora of pathways and have recently been adopted in organoids. Two recent studies have utilized this method in both Vero cells and hepatoma cells and identified host factors ACE2, the protease Cathepsin L, and TMEM106B to play a crucial role in viral infection (Wang et al., 2020b; Wei et al., 2020). Another study utilized genome-wide CRISPR screens to elucidate the cytotoxicity pathways of remdesivir, a drug that is now commonly administered to SARS-CoV-2 patients (Akinci et al., 2020). These insights gathered from classical cell lines, await confirmation in more physiologically relevant systems, such as organoids. Recently, two independent studies described the first genome-wide CRISPR screens in organoids, paving the way toward genome-wide CRISPR screens in organoids to elucidate SARS-CoV-2 biology (Michels et al., 2020; Ringel et al., 2020).

## Testing and Discovering COVID-19 Therapeutics on Organoid Platforms: Current Possibilities and the Road Ahead

Organoids are an emerging tool for predicting drug efficacy and drug discovery that are amenable to high-throughput drug screens. Tumor-derived organoids can, for example, predict the success of therapeutic regimes in corresponding cancer patients (Ooft et al., 2019). Similarly, intestinal organoids from patients suffering from the genetic disease cystic fibrosis can be employed to select effective drugs (Dekkers et al., 2013). Organoids are proving their value in testing the efficacy of antiviral treatments. Intestinal organoids infected with noroviruses were exposed to different blocking antibodies to identify those that slow viral spreading (Alvarado et al., 2018). Cerebral organoids have similarly provided a platform to identify new drugs capable of mitigating Zika virus-induced cell death in neural progenitors (Qian et al., 2017; Watanabe et al., 2017). In the past months, multiple studies have applied organoids to predict clinical success of SARS-CoV-2 treatments. Upon viral infection, cells may upregulate many different ISGs as defense mechanisms. One of these ISGs, cholesterol 25-hydroxylase, was identified upon SARS-CoV-2 infection in organoids and COVID-19 patients and is known to be effective in combating spreading of multiple other viruses. This enzyme produces 25-hydroxycholesterol from cholesterol. Treatment of lung organoids with 25-hydroxycholesterol greatly diminished SARS-CoV-2 replication, potentially through alternating the composition of cellular membranes and preventing fusion of the viral envelope with cells (Wang et al., 2020a, 2020b). A mouse-adapted strain of SARS-CoV-2, known as MASCp6, is effectively inhibited by the cholesterol derivative, in line with the organoid experiments (Zu et al., 2020). 25-Hydroxycholesterol does not have known toxicities at these concentrations and thus appears to be a therapeutic candidate for COVID-19. Other studies subjected iPSC-derived intestinal organoids and adult human bronchial organoids to multiple drugs currently tested in clinical settings, including the adenosine analog remdesivir, the histamine-2 blocker famotidine, and the TMPRSS2 inhibitor camostat (Krüger et al., 2020; Suzuki et al., 2020). The above studies did exploit *a priori* knowledge on coronavirus biology or tested well-known viral inhibitors. Drug discovery efforts would greatly benefit from unbiased drug screens to enhance the likelihood of discovering new drugs. Most drug screens currently performed on organoids are based on morphology, fluorescent readouts, or ATP-based assays that measure cellular viability. However, coronaviruses do not necessarily induce apoptosis of epithelial cells and thus may require alternative measures of drug efficacy. An additional specific challenge for high-throughput drug screens using pathogenic viruses, such as SARS-CoV-2, include the required biosafety level, which could complicate the use of robotics. A recent study used pseudoviruses (lowering handling risk) containing SARS-CoV-2 spike protein to perform a drug screen in iPSC-derived lung organoids in a 384-well format. An integrated luciferase reporter was used as a measure of viral entry and replication (Han et al., 2020). This work revealed multiple FDA-approved drugs, including the Bcr-Abl tyrosine kinase inhibitor imatinib and the immunosuppressant mycophenolic acid to be effective in lowering viral replication. A separate study using human iPSC-derived colorectal organoids utilized a similar setup and tested more than 1,000 drugs (Duan et al., 2020). Many of these hits overlapped with those in lung, indicating effective viral blockade in different epithelial tissues. These studies provide the first examples of how organoids can aid in the quest for novel COVID-19 therapies by allowing medium-throughput drug screening in a physiologically relevant system. Nonetheless, these pioneering efforts in organoids all depend on pseudoviruses that at best mimic aspects of viral entry but less of downstream processes. High-throughput screens using wild-type strains require effective monitoring of viral spreading, which was recently facilitated by reverse engineering a fluorescent SARS-CoV-2 (Hou et al., 2020). However, the large coding sequences of these reporters disturb endogenous viral genes, potentially interfering with the normal viral life cycle. Alternative to this, the epithelial cells could be genetically engineered to report the expression of viral response genes, such as ISGs. Such organoids could be effective, generic tools to study viral entry and responses of any (future) pandemic virus that are amenable to high-throughput readouts. Not only would such fluorescent reporters allow efficient measurement of viral spreading, they would also discriminate infected from non-infected cells. The latter could be used for in-depth characterization of epithelial responses to the virus. We have shown recently that large biobanks of intestinal organoids can rapidly be genetically engineered to harbor fluorescent reporters of different genes (Beumer et al., 2020). COVID-19 drug testing in organoids could also focus on modulating the expression or activity of host products important for the viral replication cycle. Multiple proteins are believed to be indispensable for viral replication, including ACE2 and the proteases TMPRSS2 and CTSL. Serine protease inhibitors targeting Tmprss2 have been developed but display many off-target effects that could hamper clinical success. The fact that organoids closely resemble their corresponding native tissue makes these attractive models in an attempt to modulate the expression of key host genes. Again, fluorescent reporters could be utilized as a measure of protein abundance or localization.

## Future Perspectives for Organoid Platforms in Virology

**Figure 2:**
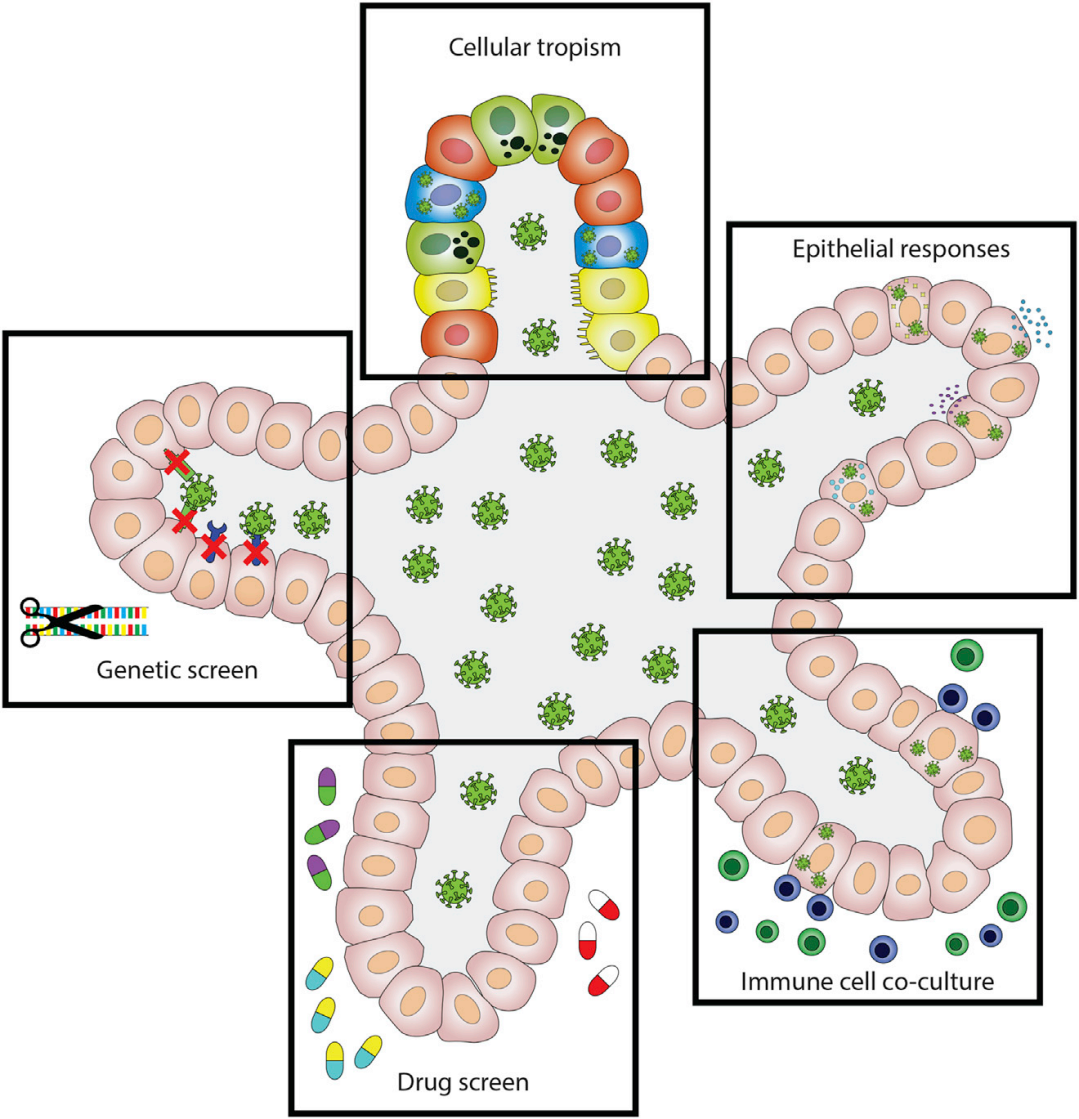
Overview of the Potential of iPSC and ASC-Derived Organoids in Virology Research

Organoids are proving useful in unraveling SARS-CoV-2 biology, including its cellular tropism. In contrast to many cell lines, human organoids can readily be infected with SARS-CoV-2 without inducing artificial expression of key host factors, such as ACE2. To increase the faithful representation of epithelial tissues, virally infected organoids could be co-cultured with other cell types. ASC-derived organoids exclusively consist of epithelial cells, while iPSC-based systems can include mesenchymal lineages, such as fibroblasts or muscle. Most importantly however, both systems lack immune cells, such as B and T cells, innate lymphoid cells, and macrophages that normally reside in mucosal tissues. All of these lineages play important executive roles in fighting viral infections, including SARS-CoV-2, as reviewed in Vabret et al. (2020). Organoids from colorectal and non-small cell lung cancer biopsies have previously been co-cultured with circulating T cells from corresponding patients. That study revealed successful antigen presentation of tumor organoid cells to T cells, which subsequently became activated (Dijkstra et al., 2018). Similar co-cultures could be applied to virus-infected organoids. We predict that further development and maturation of the organoid viral toolbox, including complex co-cultures and genetically engineered organoids, enable larger drug screens using viral strains isolated from patients. These efforts would increase the odds for deriving clinically successful COVID-19 therapeutics. Finally, organoids can be used to compare viral responses between individuals. In this review, we have discussed the current and future potential of organoids in virology research (Figure 2). Broad application of this culturing technique could contribute significantly toward eradicating COVID-19 and future viral pandemics.

## Author Contributions

M.H.G., J.v.d.V., and J.B. wrote the first draft of the manuscript. H.C. was involved in discussing and revising the contents.
